# Synthesis, Molecular Docking and Preliminary *in-Vitro* Cytotoxic Evaluation of Some Substituted Tetrahydro-naphthalene (2',3',4',6'-Tetra-*O*-Acetyl-β-D-Gluco/-Galactopyranosyl) Derivatives

**DOI:** 10.3390/molecules17044717

**Published:** 2012-04-23

**Authors:** Maha S. Al-Mutairi, Ebtehal S. Al-Abdullah, Mogedda E. Haiba, Mohammed A. Khedr, Wafaa A. Zaghary

**Affiliations:** 1 Department of Pharmaceutical Chemistry, Faculty of Pharmacy, King Saud University, Riyadh 11451, Saudi Arabia; 2 Department of Medicinal Chemistry, National Research Center, Dokki, Cairo 12622, Egypt; 3 Department of Pharmaceutical Chemistry, Faculty of Pharmacy, Helwan University, Ain Helwan, Cairo 11795, Egypt

**Keywords:** tetrahydronaphthalene, pyridine, glycoside, cytotoxic, molecular docking

## Abstract

A facile, convenient and high yielding synthesis of novel *S*-glycosides and *N*-glycosides incorporating 1,2,3,4-tetrahydronaphthalene and or 1,2-dihydropyridines moieties has been described. The aglycons **2**, **4**, and **7** were coupled with different activated halosugars in the presence of basic and acidic medium. The preliminary *in-vitro* cytotoxic evaluation revealed that compounds **3c**, **3f**, **5c** and **7b** show promising activity. A molecular docking study was performed against tyrosine kinase (TK) (PDB code: 1t46) by Autodock Vina. The docking output was analyzed and some compounds have shown hydrogen bond (H-B) formation with reasonable distances ranged from 2.06 A° to 3.06 A° with Thr 670 and Cys 673 residues found in the specified pocket. No hydrogen bond was observed with either Glu 640 nor Asp 810 residues, as was expected from pdbsum.

## 1. Introduction

Tetrahydronaphthalene derivatives have attracted significant attention in the field of drug discovery because of their wide array of pharmacological activities, including action as non-steroidal glucocorticoid receptor modulators, anticancer, analgesic, anti-inflammatory, antimicrobial, antiplatelet aggregation, hypotensive, antiarrhythmic and anti-HIV effects [1,2,3,4,5,6,7,8,9,10,11]. Additionally, pyridine analogs are very important class of heterocyclic compounds that have remarkable pharmacological activities as PDE4 inhibitors, analgesic, antifungal, antimalarial, anti-inflammatory, antibacterial, anti-HIV, antitumor and antiviral properties [12,13,14,15,16,17].

On the other hand recent advances have implicated the role of tyrosine kinases (TK) in the physiology of cancer. Though their activity is tightly regulated in normal cells, they may acquire transforming functions due to mutation(s), over expression leading to malignancy [18,19]. The activation of cancer cells can be blocked by selective tyrosine kinase inhibitors and thus considered to be a promising target for anticancer drug discovery. Tyrosine kinases are important mediators of the signal transduction process, leading to cell proliferation, differentiation, migration, metabolism and apoptosis. Tyrosine kinases are a family of enzymes, which catalyzes phosphorylation of select tyrosine residues in target proteins, using ATP. They are implicated in several steps of neoplastic development and progression. Tyrosine kinase signaling pathways normally prevent deregulated proliferation or contribute to sensitivity towards apoptotic stimuli. These signaling pathways are often genetically or epigenetically altered in cancer cells to impart a selection advantage to the cancer cells [20,21,22].

Regarding the above mentioned findings and our previous reports [23] and [24], the main goal of the present work was to design, synthesize and investigate the anticancer activity of some novel pyridine-tetrahydronaphthalene derivatives carrying carbohydrate residues attached through *S*-glycosidic or *N*-glycosidic bond formation. A molecular docking study was performed in order to interpret the activity expressed by compounds **3c**, **3f**, **5b**, **5c**, **7a** and **7b**.

## 2. Results and Discussion

### 2.1. Chemistry

**Scheme 1:**
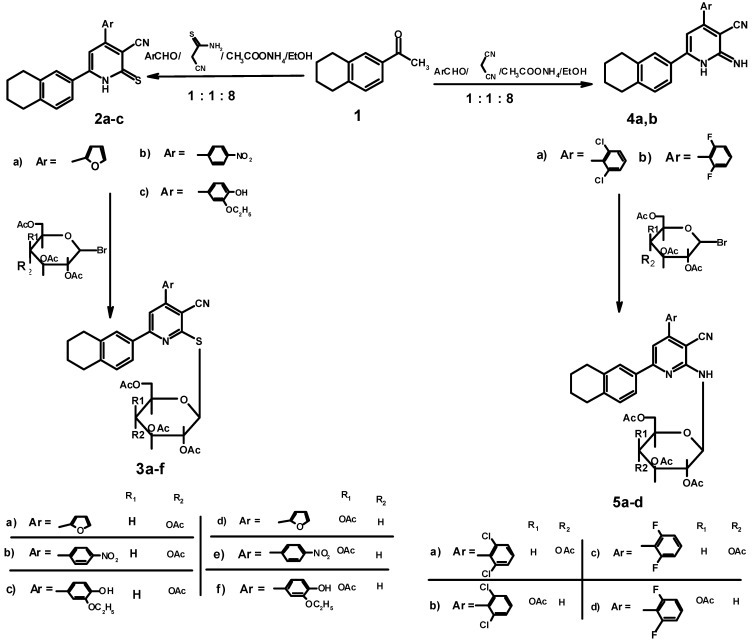
Synthetic pathways to compounds **2a–c**, **3a–f**, **4a,b** and **5a–d**.

A series of *S*-glycosides and *N*-glycosides was designed in such a way that they incorporate a tetrahydronaphthalene derivative attached to a glycosyl moiety through a *S*-linkage or *N*-linkage. Synthesis of our desired compounds was achieved by allowing the acetyl derivative 1-(1,2,3,4-tetrahydronaphthaline-6-yl)ethanone (**1**) to react directly with the appropriate aromatic aldehyde and cyanothioacetamide in the presence of ammonium acetate, to afford the corresponding 2-thioxo(1H)pyridines **2a–c**, respectively (Scheme 1, Table 1). The coupling between the aglycon and the activated cyclic sugars (2',3',4',6'-*O*-acetyl-α-D-gluco- (or galato-) pyranosyl bromide was achieved in a basic medium to give the corresponding thioglycoside derivatives **3a–f** in a good yields, respectively (Scheme 1, Table 1). The structures of thioglycosides **3a–f** were established and confirmed on the basis of their elemental analysis and spectral data (IR, ^1^H-NMR, ^13^C-NMR and MS, cf. Experimental).

**Table 1:** Physicochemical properties of the newly synthesized compounds **2a**, **3a–f**, **5a–d**, **7a**, **7b**, **8a** and **8b**.

Comp No.	Ar	R_1_	R_2_	MP (°C)	Cryst.Solv.	Yield %	Molecular Formula (Mol. Wt.)
**2a**	-C_4_H_3_O	---	---	245–247	Ethanol/H_2_O	80	C_20_H_16_N_2_OS (332)
**3a**	-C_4_H_3_O	H	OAc	194–196	Ethanol/H_2_O	76	C_34_H_34_N_2_O_10_S (662)
**3b**	4-NO_2-_C_6_H_5_	H	OAc	172–174	Ethanol/H_2_O	75	C_36_H_35_N_3_O_11_S (717)
**3c**	3-EtO,4-OH-C_6_H_4_	H	OAc	118–120	Ethanol/H_2_O	68	C_38_H_40_N_2_O_11_S (732)
**3d**	-C_4_H_3_O	OAc	H	215–217	Ethanol/H_2_O	75	C_34_H_34_N_2_O_10_S (662)
**3e**	4-NO_2-_C_6_H_5_	OAc	H	140–142	Ethanol/H_2_O	76	C_36_H_35_N_3_O_11_S (717)
**3f**	3-EtO-4-OH-C_6_H_4_	OAc	H	175–177	Ethanol/H_2_O	79	C_38_H_40_N_2_O_11_S (732)
**5a**	2,6-Cl_2_-C_6_H_4_	H	OAc	192–194	Ethanol/H_2_O	65	C_36_H_35_Cl_2_N_3_O_9_ (724)
**5b**	2,6-Cl_2_-C_6_H_4_	OAc	H	205–207	Ethanol/H_2_O	70	C_36_H_35_Cl_2_N_3_O_9_ (724)
**5c**	2,6-F_2_-C_6_H_4_	H	OAc	107–109	Ethanol/H_2_O	66	C_36_H_35_F_2_N_3_O_9_ (691)
**5d**	2,6-F_2_-C_6_H_4_	OAc	H	132–134	Ethanol/H_2_O	72	C_36_H_35_F_2_N_3_O_9_ (691)
**7a**	2,6-Cl_2_-C_6_H_4_	---	---	180–182	Ethanol/H_2_O	95	C_22_H_18_C_l2_N_2_O_2_ (413)
**7b**	2,6-F_2_-C_6_H_4_	---	---	170–172	Ethanol/H_2_O	92	C_22_H_18_F_2_N_2_O_2_ (380)
**8a**	2,6-Cl_2_-C_6_H_4_	OAc	H	230–232	Ethanol/H_2_O	86	C_36_H_36_Cl_2_N_2_O_11_ (743)
**8b**	2,6-F_2_-C_6_H_4_	OAc	H	155–157	Ethanol/H_2_O	85	C_36_H_36_F_2_N_2_O_11_ (710)

Also, one-step synthesis of 2-imino (1H)pyridines **4a,b** was accomplished by heating the acetyl derivative **1** with malononitrile and the appropriate aldehyde in the presence of ammonium acetate (Scheme 1, Table 1). The authors used these intermediate aglycones for the preparation of the new glycosides **5a–d** (Scheme 1, Table 1). The formation of *N*-glycosides **5a–d** was proven using spectral data (IR, ^1^H-NMR, ^13^C-NMR and MS).

**Scheme 2:**
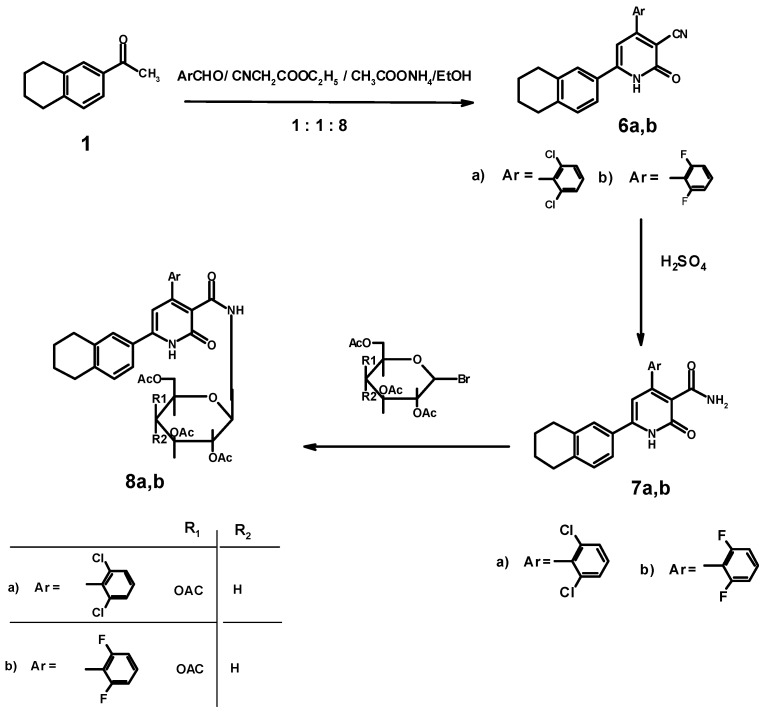
Synthetic pathways to compounds **6a,b**, **7a,b** and **8a,b**.

On the other hand, the one pot reaction of compound **1** with the appropriate aromatic aldehyde and ethyl cyanoacetate in the presence of ammonium acetate, afforded the corresponding pyridones **6a,b**, respectively (Scheme 2, Table 1). Hydrolysis of **6a,b** in the presence of conc. sulphuric acid afforded the amide derivatives **7a,b** in good yield. Finally the coupling between the aglycons **7a,b** and the activated cyclic sugar 2',3',4',6'-tetra-*O*-α-D-galactopyranosyl bromide gave the corresponding *N*-glycosides **8a,b** in a good yield respectively. The structures of *N*-glycosides **8a,b** were established and confirmed for the reaction products on the basis of their spectral data.

### 2.2. Molecular Modeling Study

#### Docking Using Autodock Vina

**Figure 1:**
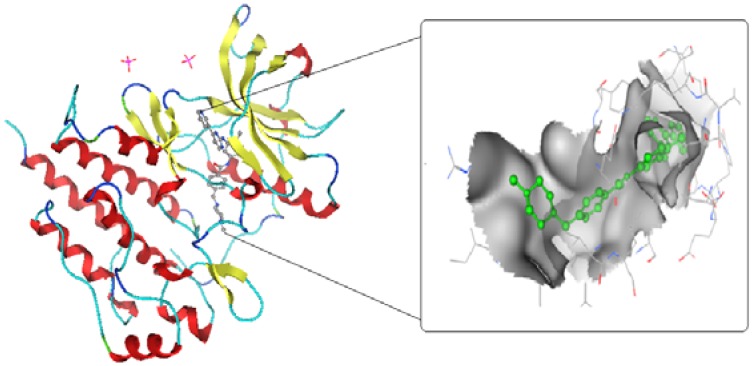
Showing the active site at which the inhibitor complexed.

**Figure 2:**
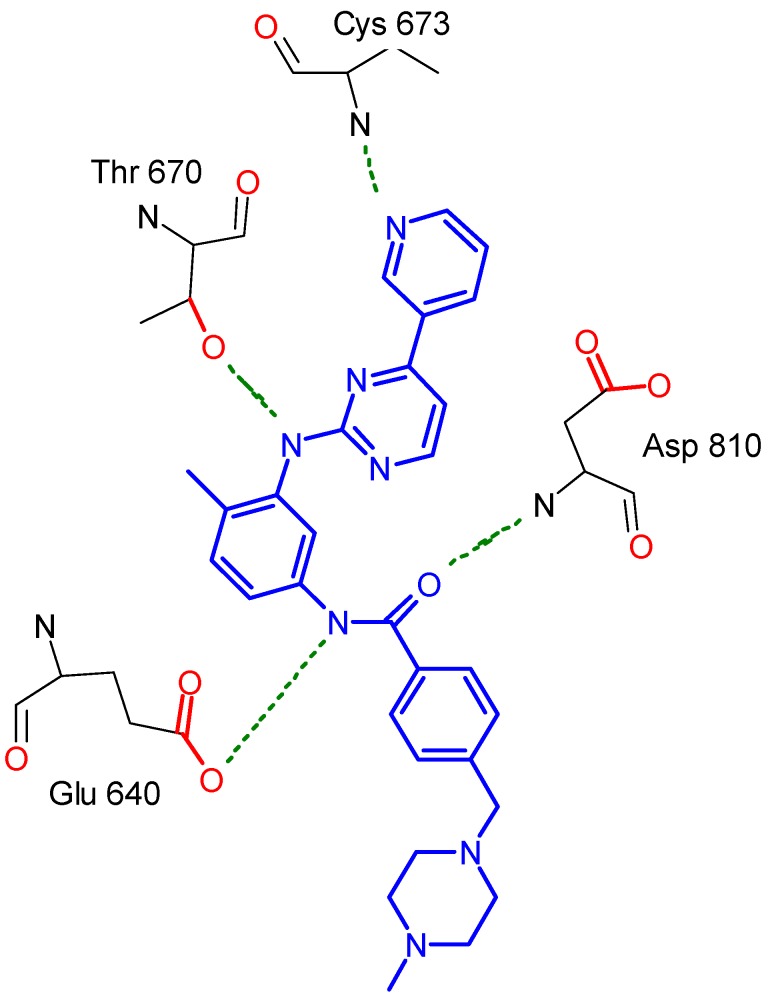
A representation image simulating the interactions that was found in pdbsum, showing the most visible interactions that were formed with the complexed ligand.

The tyrosine kinase (TK) crystal structure (PBD code = 1t46) was downloaded (Figure 1). In order to find the important residues that form interactions with that inhibitor, the pdbsum server was used. It was found that Cys 673, Thr 670, Glu 640 and Asp 810 formed some interactions with different moieties of the inhibitor (Figure 2).

The affinity of any small molecule can be considered to be a unique tool in the field of drug design, as there is a relationship between the affinity of organic molecules and the free binding energy and this can contribute in prediction and interpretation of the activity of organic compounds toward a specific target protein. Here we used the Autodock Vina [25,26] program for docking and to obtain the affinities and RMSD (root mean square deviation) of all compounds.

The most visible feature that was observed in the docking output that all compounds fitted well inside the same docking site and hence, the next step was to compare their affinities and try to rank them as observed in the previous table and then to measure the ligands’ interaction to find out which ones interact well and correlate that to the activity.

The six compounds with the highest calculated affinities were found to be compounds **7b**, **3f**, **5c**, **5b**, **3c** and **7a**, respectively (−11.6, −11.4, −11.2, −11.0, −10.7 and −10.3 Kcal/mol). Compounds **3c** and **7b** were found to have low RMSD values that reflect their better fit as they were expected to have a conformation with a low deviation from the expected best binding mode. No clear correlation between the biological activity and the calculated affinity was observed. The only observation that was found that the active compounds have the best affinities regardless of their ranking in activity.

**Figure 3:**
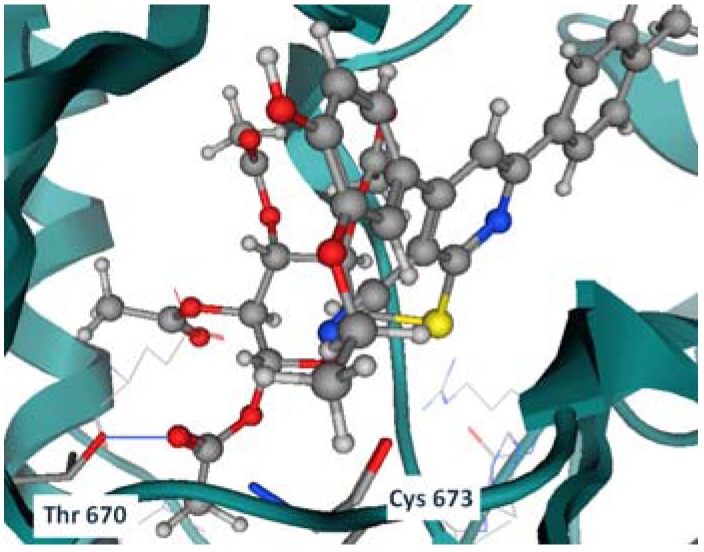
Compound **3c** showing hydrogen bond with Thr 670 with the -CH_3_CO of the compound.

Regarding the best mode of interaction for each compound of these six compounds, the docking output was visualized to determine these interactions. It was important to concentrate on the previously mentioned residues (Cys 673, Thr 670, Glu 640 and Asp 810) whether they were involved in these interactions or not. For compound **3c** THR 670 (Figure 3) was the major residue for making hydrogen bond with one of the acetyl groups found in the side chains of this compound.

**Figure 4:**
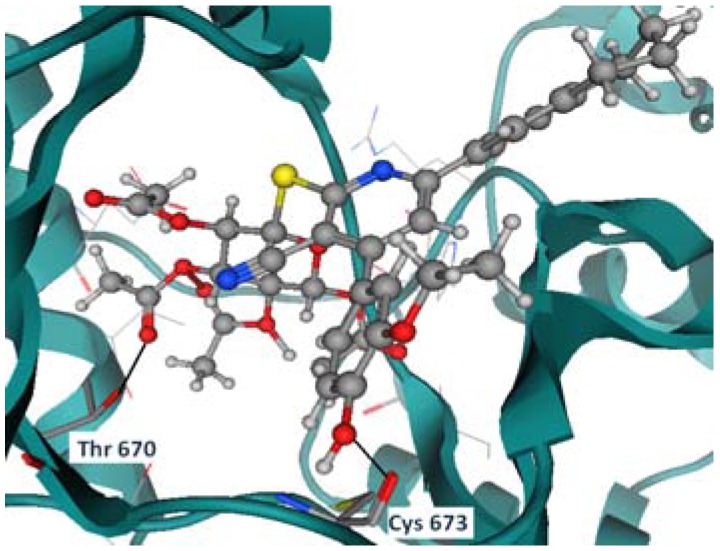
Compound **3f** hydrogen bond formation with Thr 670 and Cys 673.

**Figure 5:**
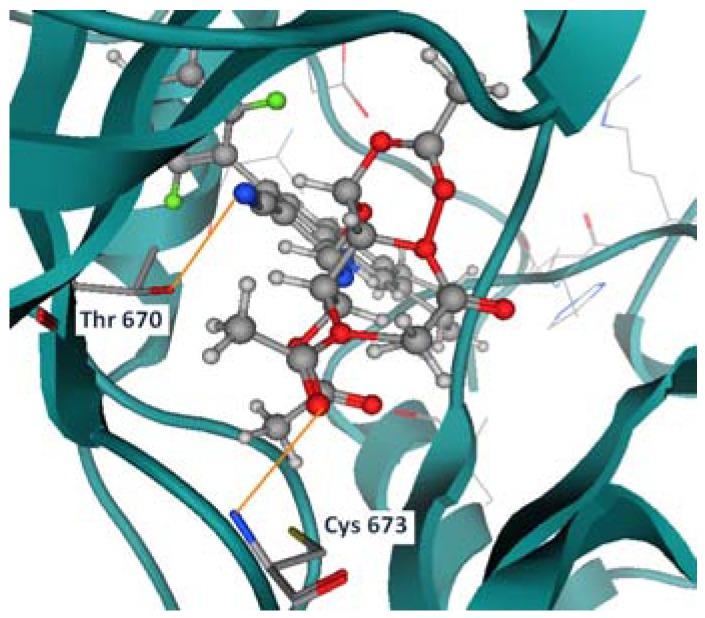
Compound **5c** hydrogen bond formation with Thr 670 and Cys 673.

**Figure 6:**
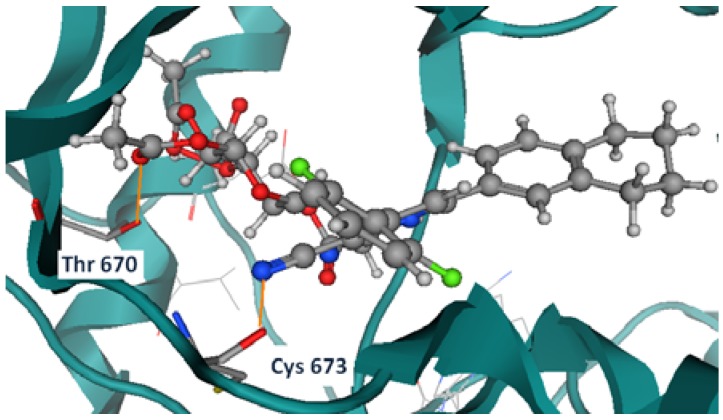
Compound **5b** hydrogen bond formation with Thr 670 and Cys 673.

**Figure 7:**
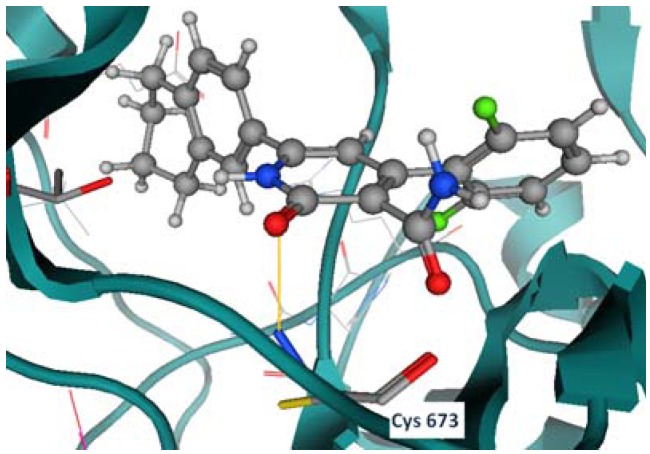
Compound **7b** hydrogen bond formation with Cys 673.

Compound **3f** shows two hydrogen bonds with Thr 670 and Cys 673 (Figure 4). Compounds **5c**, **5b**, and **7b** have shown the same hydrogen bond with the previously mentioned residues as well (Thr 670, and Cys 673), as shown in Figure 5, Figure 6, Figure 7.

### 2.3. *In Vitro* Cytotoxic Screening

Some of the newly synthesized compounds were tested at the Department of Tumor Pathology, National Cancer Institute, Cairo, Egypt. Ehrlich Ascites Carcinoma (EAC) cells were used for cytotoxic activity screening. The selected synthesized analogues **2b**, **3a–f**, **5b**, **5c**, **7a** and **7b** were tested for *in vitro* cytotoxic activity at two different concentrations. The results (Table 2) are expressed in the form of the percentage of non-viable cells [27]. The *in vitro *evaluation revealed that **3c**, **3f**, **5c** and **7b** show promising activity. This provides a new class of antitumor agents for further biological evaluation.

**Table 2:** The effect of the tested compounds on the viability of tumor cell *in vitro*. The calculated affinities and RMSD for some of the newly synthesized compounds.

Compound	Anticancer effect % inhibition of cell viability		Affinity Kcal/mol	RMSD Deviation from best mode
	100 µg/mL	50 µg/mL		
**2b**	0	0	−9.5	7.07
**3a**	0	0	−8.3	9.5
**3b**	0	0	−7.7	11.6
**3c**	70%	50%	−10.7	4.02
**3d**	0	0	−6.6	10.3
**3e**	0	0	−7.4	9.2
**3f**	10%	0	−11.4	9.0
**5b**	0	0	−11.0	9.3
**5c**	40%	7%	−11.2	9.5
**7a**	0	0	−10.3	7.2
**7b**	20%	15%	−11.6	4.6

## 3. Experimental

### 3.1. Chemistry

#### 3.1.1. General

Melting points (°C, uncorrected) were determined in open glass capillaries using a Barnstead 9001 Electrothermal melting point apparatus. Infrared (IR) spectra were recorded on a Perkin Elmer FT-IR Spectrum BX Spectrometer at cm^−1^ scale using KBr discs. ^1^H-NMR and ^13^C-NMR were recorded on a JEOL 300 MHz Spectrometer; chemical shifts are expressed in δ (ppm) with reference to TMS. The mass spectra were run at 70 ev with Packard 5890 Thewlett GC/MS Spectrometer, using the Electron Ionization (EI) technique. Elemental analyses (C, H, N) were carried out at the Microanalytical Data Center, Faculty of Science, Cairo, Egypt, and were in full agreement with the proposed structures within ±0.4% of the theoretical values. Thin layer chromatography was performed on precoated (0.75 mm) silica gel GF254 plates (E. Merck, Germany). Visualization was performed by illumination with UV light source (254 nm). Compounds **2b**, **2c**, **4a**, **4b**, **6a** and **6b** were prepared using the previously published procedures [10] and [11] as described below (Scheme 1 and Scheme 2).

#### 3.1.2. Synthesis of *4-Aryl-6-(1,2,3,4-tetrahydronaphthalen-6-yl)-2-thioxo-1,2-dihydropyridine-3-carbonitriles* **2a–c**

A mixture of 6-acetyltetraline **1** (0.3 g, 0.0017 mol), 2-cyanothioacetamide (0.17 g, 0.0017 mol), the an appropriate aromatic aldehyde, namely 2-furfural, *p*-nitrobenzaldehyde, and/or 3-ethoxy-4-hydroxybenzaldehyde (0.00172 mol) and excess ammonium acetate (1.05 g, 0.0137 mol) was gently refluxed in absolute ethanol (15 mL) for 3–8 h. On cooling, the separated solid was filtered off, washed successively with water, and then with ether and recrystallized with aqueous ethanol to give **2a–c** respectively (Table 1).

*4-(Furan-2-yl)-6-(1,2,3,4-tetrahydronaphthalen-6-yl)-2-thioxo)-1,2-dihydropyridine-3-carbonitrile* (**2a**). IR: (υ, cm^−1^) 3,300 (NH), 2,200 (CN) and 1,206 (C-O). ^1^H-NMR (DMSO-d_6_): δ 1.76 (m, 4H, -CH_2_-CH_2_-), 2.78 [m, 4H, 2(CH_2_)] attached to the aromatic ring of tetrahydronaphthalene), 6.89–8.61 (m, 7H, aromatic protons & pyridine-H) and 13.9 (NH). ^13^C-NMR (DMSO-d_6_): δ 22.9 (-CH_2_-CH_2_-), 29.2 [2(CH_2_)] attached to the aromatic ring of tetrahydronaphthalene), 114.2 (CN), 106.2, 107.6, 117.7, 117.9, 125.66, 128.7, 129.3, 129.8, 137.8, 141.5, 142.1, 147.5, 148.3, 152.6, 180.4 (Ar-C), MS, *m/z* (%): (M^+^, 332, 0.05%).

#### 3.1.3. Synthesis of *4-Aryl-6-(1,2,3,4-tetrahydronaphthalen-6-yl)-2-(2',3',4',6'-tetra-O-acetyl-β-D-gluco and galactopyranosyl thio)pyridine-3-carbonitriles* **3a–f**

A mixture of **2a–c** (0.01 mol) in ethanol (10 mL) and potassium hydroxide (0.56 g, 0.01 mol) in distilled water (6 mL) was added to a solution of 2,3,4,6-tetra-*O*-acetyl-α-D-gluco- or galactopyranosyl bromide (4.10 g, 0.01 mol) in acetone (30 mL). The reaction mixture was stirred at room temperature until completion (TLC) 20–24 h. and then the solution was evaporated and poured onto ice/cold water. The formed solid product was collected by filtration. The residue was washed with distilled water to remove the formed potassium bromide. The resulting solid product was dried and crystallized from dilute ethanol (Table 1).

*4-(Furan-2-yl)-6-(1,2,3,4-tetrahydronaphthalen-6-yl)-2-(2',3',4',6'-tetra-O-acetyl-β-D-glucopyranosylthio)pyridine-3-carbonitrile* (**3a**). IR: (υ, cm^−1^) 2937 (CH, alkane), 2,216 (CN), 1,748 (C=O) and 1,230 (C-O). ^1^H-NMR (DMSO-d_6_): δ 1.78 (m, 4H, -CH_2_-CH_2_-), 2.02–2.03 (4s, 12H, 4 × CH_3_CO), 2.88 [m, 4H, 2(CH_2_)] attached to the aromatic ring of tetrahydronaphthalene), 4.02–4.06 (m, 2H, 6'-H_2_) 4.31 (m, 1H, 5'-H), 5.03 (t, 1H, 4'-H) 5.26 (t, 1H, 3'-H), 5.71 (t, 1H, 2'-H), 6.24 (d, 1H, 1'-H) and 6.84–8.13 (m, 7H, aromatic protons and pyridine-H). ^13^C-NMR (DMSO-d_6_): δ 22.9 (-CH_2_-CH_2_-), 29.2 [2(CH_2_)] attached to the aromatic ring of tetrahydronaphthalene), 115.7 (CN), 62.29 (CH_2_, C-6'), 68.20 (C-4'), 69.76 (C-2'), 73.1 (C-3'), 75.17 (C-5'), 83.90 (C-1'), 106–180 (Ar-C) and 169.5–170.3 (4 × CO). MS, *m/z* (%): (M^+^, 662, 0.03%).

*4-(4-Nitrophenyl)-6-(1,2,3,4-tetrahydronaphthalen-6-yl)-2-(2',3',4',6'-tetra-O-acetyl-β-D-glucopyranosylthio)pyridine-3-carbonitrile* (**3b**). IR: (υ, cm^−1^) 2,930 (CH, alkane), 2,217 (CN), 1,752 (C=O) and 1,348 & 1,528 (NO_2_). ^1^H-NMR (DMSO-d_6_): δ 1.75 (m, 4H, -CH_2_-CH_2_-), 2.04 (4s, 12H, 4 × CH_3_CO), 2.76 [m, 4H, 2(CH_2_)] attached to the aromatic ring of tetrahydronaphthalene), 4.04–4.06 (m, 2H, 6'-H_2_) 4.35 (m, 1H, 5'-H), 5.05 (t, 1H, 4'-H) 5.24 (t, 1H, 3'-H), 5.74 (t, 1H, 2'-H), 6.31 (d, 1H, 1'-H) and 7.1–8.4 (m, 8H, aromatic protons and pyridine-H). ^13^C-NMR (DMSO-d_6_): δ 22.18 (-CH_2_-CH_2_-), 19.62–19.90 (4 × -COCH_3_), 28.65 [2(CH_2_)] attached to the aromatic ring of tetrahydronaphthalene), 61.5 (CH_2_, C-6'), 67.2 (C-4'), 69.5 (C-2'), 72.9 (C-3'), 74.6 (C-5'), 79.6 (C-1'), 115.46 (CN), 102–147 (Ar-C), 169.5 (4 × CO).MS, *m/z* (%): (M^+^, 717, 1.34%).

*4-(3-Ethoxy-4-hydroxyphenyl)-6-(1,2,3,4-tetrahydronaphthalen-6-yl)-2-(2',3',4',6'-tetra-O-acetyl-β-D-glucopyranosyl thio)pyridine-3-carbonitrile* (**3c**). IR: (υ, cm^−1^) 3,458 (OH), 2,937 (CH, alkane), 2,216 (CN), 1,754 (C=O) and 1,125 (C-O). ^1^H-NMR (DMSO-d_6_): δ 1.36 (t, 3H, OCH_2_CH_3_), 1.78 (m, 4H, -CH_2_-CH_2_-), 1.9–2.03 (4s, 12H, 4 × CH_3_CO), 2.87 [m, 4H, 2(CH_2_)] attached to the aromatic ring of tetrahydronaphthalene), 4.01–4.35 (m, 5H, 6'-H_2_, 5'-H & OCH_2_CH_3_), 4.9 (t, 1H, 4'-H) 5.2 (t, 1H, 3'-H), 5.7 (t, 1H, 2'-H), 6.29 (d, 1H, 1'-H) 6.94–8.1 (m, 7H, aromatic protons & pyridine-H) and 9.6 (OH). ^13^C-NMR (DMSO-d_6_): δ 14.49 (-OCH_2_CH_3_), 20.28 (4 × -COCH_3_), 22.49 (-CH_2_-CH_2_-), 28.63 [2(CH_2_)] attached to the aromatic ring of tetrahydronaphthalene), 61.54 (CH_2_, C-6'), 63.89 (-O-CH_2_-CH_3_), 67.52 (C-4'), 69.50 (C-2'), 72.89 (C-3'), 74.63 (C-5'), 79.60 (C-1'), 115.75 (CN), 113–158 (Ar-C) and 169.45 (4 × CO). MS, *m/z* (%): (M^+^, 732, 12.15%).

*4-(Furan-2-yl)-6-(1,2,3,4-tetrahydronaphthalen-6-yl)-2-(2',3',4',6'-tetra-O-acetyl-β-D-galactopyranosyl thio)pyridine-3-carbonitrile* (**3d**). IR: (υ, cm^−1^) 2,936 (CH, alkane), 2,216 (CN), 1,754 (C=O) and 1,239 (C-O). ^1^H-NMR (DMSO-d_6_) δ 1.78 (m, 4H, -CH_2_-CH_2_-), 1.96–2.14 (4s, 12H, 4 × CH_3_CO), 2.88 [m, 4H, 2(CH_2_)] attached to the aromatic ring of tetrahydronaphthalene), 3.95 (m, 2H, 6'-H_2_), 4.10 (m, 1H, 5'-H), 4.53 (t, 1H, 4'-H) 5.25 (t, 1H, 3'-H), 5.41 (t, 1H, 2'-H), 6.22 (d, 1H, 1'-H), 6.8–8.1 (m, 7H, aromatic protons and pyridine-H). ^13^C-NMR (DMSO-d_6_): δ 22.9 (-CH2-CH2-), 29.2 (2(CH_2_) attached to the aromatic ring of tetrahydronaphthalene), 115.7 (CN), 61.70 (CH2, C-6'), 67.74 (C-4'), 69.45 (C-2'), 72.64 (C-3'), 74.97 (C-5'), 84.74 (C-1'), 106–180 (Ar-C) and 169.03–169.87 (4 × CO). MS, *m/z* (%): (M^+^, 662, 0.71%).

*4-(4-Nitrophenyl)-6-(1,2,3,4-tetrahydronaphthalen-6-yl)-2-(2',3',4',6'-tetra-O-acetyl-β-D-galactopyranosyl thio)pyridine-3-carbonitrile* (**3e**). IR: (υ, cm^−1^) 2,932 (CH, alkane), 2,217 (CN), 1751 (C=O) and 1,348 & 1,528 (NO_2_). ^1^H-NMR (DMSO-d_6_): δ 1.76 (m, 4H, -CH_2_-CH_2_-), 1.97–2.14 (4s, 12H, 4 × CH_3_CO), 2.77 [m, 4H, 2(CH_2_)] attached to the aromatic ring of tetrahydronaphthalene), 3.94 (m, 2H, 6'-H_2_), 4.08 (m, 1H, 5'-H), 4.55 (t, 1H, 4'-H) 5.30 (t, 1H, 3'-H), 5.40 (t, 1H, 2'-H), 6.25 (d, 1H, 1'-H) and 7.21–8.43 (m, 8H, aromatic protons and pyridine-H). ^13^C-NMR (DMSO-d_6_): δ 22.2 (-CH_2_-CH_2_-), 19.6–19.9 (4 × -COCH_3_), 28.82 (2(CH_2_) attached to the aromatic ring of tetrahydronaphthalene), 61.28 (CH_2_, C-6'), 65.23 (C-4'), 66.67 (C-2'), 71.34 (C-3'), 74.46 (C-5'), 80.75 (C-1'), 115.46 (CN), 102–147 (Ar-C), 169.56–169.70 (4 × -CO of COCH_3_ groups). MS, *m/z* (%): (M^+^ −1,716, 8.9%).

*4-(3-Ethoxy-4-hydroxyphenyl)-6-(1,2,3,4-tetrahydronaphthalen-6-yl)-2-(2',3',4',6'-tetra-O-acetyl-β-D-galactopyranosyl thio)pyridine-3-carbonitrile* (**3f**). IR: (υ, cm^−1^) 3,458 (OH), 2,217 (CN), 1,752 (C=O) and 1,056 (C-O). ^1^H-NMR (DMSO-d_6_): δ 1.39 (t, 3H, OCH_2_CH_3_), 1.80 (m, 4H, -CH_2_-CH_2_-), 1.96–2.06 (4s, 12H, 4 × CH_3_CO), 2.79 [m, 4H, 2(CH_2_)] attached to the aromatic ring of tetrahydronaphthalene), 4.08 (m, 2H, 6'-H_2_) 4.11 (m, 1H, 5'-H), 4.13 (t, 2H, -OCH_2_CH_3_) 4.5 (t, 1H, 4'-H) 5.26–5.65 (t, 2H, 3'-H & 2'-H), 6.25 (d, 1H, 1'-H) and 6.98–8.08 (m, 7H, aromatic protons and pyridine-H). ^13^C-NMR (DMSO-d_6_): δ 13.96 (-OCH_2_CH_3_) 19.62–19.85 (4 × -COCH_3_), 22.17 (-CH_2_-CH_2_-), 28.81 [2(CH_2_)] attached to the aromatic ring of tetrahydronaphthalene), 61.25 (CH_2_, C-6'), 64.06 (-O-CH_2_-CH_3_), 65.27 (C-4'), 66.73 (C-2'), 71.40(C-3'), 74.34 (C-5'), 80.69 (C-1'), 115.6 (CN), 110–147 (Ar-C) and 168.56–169.52 (4 × CO). MS, *m/z* (%): (M^+^, 732, 6.9%).

#### 3.1.4. Synthesis of *4-Aryl-6-(1,2,3,4-tetrahydronaphthalen-6-yl)-2-(2',3',4',6'-tetra-O-acetyl-β-D-gluco and galactopyranosyl imino)pyridine-3-carbonitriles* **5a–d**

To a solution of **4a** and/or **4b** (0.001 mol) in acetone (10 mL), 2,3,4,6-tetra-*O*-acetyl-α-D-gluco- or galactopyranosyl bromide (4.10 g, 0.01 mol) was added. A few drops of acetic acid were added and the reaction mixture was heated with stirring on a water bath at 40–50 °C for 25 h. The reaction mixture was cooled and then poured onto ice water. The formed precipitate was washed for several times with water and recrystallized from aqueous ethanol (Table 1).

*4-(2,6-Dichlorophenyl)-6-(1,2,3,4-tetrahydronaphthalen-6-yl)-2-(2',3',4',6'-tetra-O-acetyl-β-D-gluco-pyranosyl imino)pyridine-3-carbonitrile* (**5a**). IR: (υ, cm^−1^) 3,450 (NH), 2,990 (CH_2_), 2,216 (CN), 1,750 (C=O). ^1^H-NMR (DMSO-d_6_): δ 1.79 (m, 4H, tetrahydronaphthalene CH_2_), 1.98–2.29 (4s, 12H, 4 × CH_3_CO) 2.81 (m, 4H, tetrahydronaphthalene CH_2_), 4.09 (s, 2H, 6'-H), 4.54 (m, 1H, 5'-H), 4.64 (m, 2H, 4'-H, 3'-H), 5.46 (t, 1H, 2'-H), 5.95 (d, 1H, 1'H), 7.14–7.84 (m, 7H, Ar-H and pyridine-H). ^13^C-NMR (DMSO-d_6_): 20.65–20.87 (4 × -COCH_3_), δ 22.2 (-CH_2_-CH_2_-), 28.7 (2(CH_2_) attached to the aromatic ring of tetrahydronaphthalene), 61.9 (CH_2_, C-6'), 68.4 (C-4'), 68.8 (C-2'), 73.9 (C-3'), 75.25 (C-5'), 81.9 (C-1'), 115 (CN), 106–176 (Ar-C & pyridine-C), 169.5–170.3 (4 × CO). MS, *m/z* (%): (M^+^ +1, 725, 4.47%).

*4-(2,6-Dichlorophenyl)-6-(1,2,3,4-tetrahydronaphthalen-6-yl)-2-(2',3',4',6'-tetra-O-acetyl-β-D-galactopyranosyl imino)pyridine-3-carbonitrile* (**5b**). IR: (υ, cm^−1^) 3,360 (NH), 2,929 (CH_2_), 2,212 (CN), 1,737 (C=O). ^1^H-NMR (DMSO-d_6_): δ 1.74 (m, 4H, tetrahydronaphthalene CH_2_), 2–2.01 (4s, 12H, 4 × CH_3_CO) 2.76 (m, 4H, tetrahydronaphthalene CH_2_), 4.01–4.06 (s, 2H, 6'-H), 4.51 (m, 1H, 5'-H), 4.71 (t, 1H, 4'), 5.47 (t, 1H, 3'-H), 5.62 (t, 1H, 2'-H), 6.53 (d, 1H, 1'H), 7.14–7.84 (m, 7H, Ar-H and pyridine-H). ^13^C-NMR (DMSO-d_6_): 20.69–19.89 (4 × -COCH_3_), 22.17 (-CH_2_-CH_2_-), 28.81 (2(CH_2_) attached to the aromatic ring of tetrahydronaphthalene), 61.94 (CH_2_, C-6'), 66.22 (C-4'), 68.01 (C-2'), 71.73(C-3'), 74.33 (C-5'), 82.28 (C-1'), 115.6 (CN), 111–180 (Ar-C & pyridine C) and 169.56–170.33(4 × CO).MS, *m/z* (%): (M^+^ +1, 725, 3%).

*4-(2,6-Difluorophenyl)-6-(1,2,3,4-tetrahydronaphthalen-6-yl)-2-(2',3',4',6'-tetra-O-acetyl-β-D-glucopyranosyl imino)pyridine-3-carbonitrile* (**5c**).IR: (υ, cm^−1^) 3,359 (NH), 2,932 (CH_2_), 2,214 (CN), 1,750 (C=O). ^1^H-NMR (DMSO-d_6_): δ 1.75 (m, 4H, tetrahydronaphthalene CH_2_), 2–2.02 (4s, 12H, 4 × CH_3_CO) 2.77 (m, 4H, tetrahydronaphthalene CH_2_), 4.02 (s, 2H, 6'-H), 4.18 (m, 1H, 5'-H), 4.70 (t, 1H, 4'), 4.92 (t, 1H, 3'-H), 5.25 (t, 1H, 2'-H), 6.62 (d, 1H, 1'H), 7.15–8.55 (m, 7H, Ar-H and pyridine-H). MS, *m/z* (%):^13^C-NMR (DMSO-d_6_): 20.65–20.87 (4 × -COCH_3_), δ 22.4 (-CH_2_-CH_2_-), 28.91 (2(CH_2_) attached to the aromatic ring of tetrahydronaphthalene), 61.9 (CH_2_, C-6'), 68.4 (C-4'), 68.8 (C-2'), 73.9 (C-3'), 75.25 (C-5'), 81.9 (C-1'), 115.8 (CN), 111.52–174.1 (Ar-C and pyridine-C), 169.5–170.3 (4 × CO). MS, *m/z* (%): (M^+^, 691, 16%).

*4-(2,6-Difluorophenyl)-6-(1,2,3,4-tetrahydronaphthalen-6-yl)-2-(2',3',4',6'-tetra-O-acetyl-β-D-galactopyranosyl imino)pyridine-3-carbonitrile* (**5d**). IR: (υ, cm^−1^) 3,430 (NH), 2,990 (CH_2_), 2,217 (CN), 1,750 (C=O). ^1^H-NMR (DMSO-d_6_): δ 1.75 (m, 4H, tetrahydronaphthalene CH_2_), 1.99–2.01 (4s, 12H, 4 × CH_3_CO) 2.76 (m, 4H, tetrahydronaphthalene CH_2_), 4.05 (s, 2H, 6'-H), 4.2 (m, 1H, 5'-H), 4.68 (t, 1H, 4'), 4.95 (t, 1H, 3'-H), 5.37 (t, 1H, 2'-H), 6.65 (d, 1H, 1'H), 7.15–9.54 (m, 7H, Ar-H and pyridine-H). ^13^C-NMR (DMSO-d_6_): 20.69–19.89 (4 × -COCH_3_), 22.17 (-CH_2_-CH_2_-), 28.81 (2(CH_2_) attached to the aromatic ring of tetrahydronaphthalene), 61.94 (CH_2_, C-6'), 66.22 (C-4'), 68.01 (C-2'), 71.73(C-3'), 74.33 (C-5'), 82.28 (C-1'), 115 (CN), 111–176 (Ar-C and pyridine C) and 169.56–170.33 (4 × CO). MS, *m/z* (%): (M^+^, 691, 2%).

#### 3.1.5. Synthesis of *4-Aryl-6-(1,2,3,4-tetrahydronaphthalen-6-yl)-2-oxo-1,2-dihydropyridine-3-carboxamides* **7a,b**

A mixture of **6a,b** (0.01 mol) and conc. sulphuric acid (15 mL) was stirred at room temperature 6 h, and then poured on cold water. The formed solid product was collected by filtration and recrystallized from ethanol to give **7a,b** (Table 1).

*4-(2,6-Dichlorophenyl-6-(1,2,3,4-tetrahydronaphthalen-6-yl)-2-oxo)-1,2-dihydropyridine-3-carboxamide* (**7a**). IR: (υ, cm^−1^) br. 3,382 (NH & NH_2_), 2,931 (CH_2_) and 1,668 (C=O). ^1^H-NMR (DMSO-d_6_): δ 1.69 (m, 4H, -CH_2_-CH_2_-), 2.70 [m, 4H, 2(CH_2_)] attached to the aromatic ring of tetrahydronaphthalene), 4.03 (br. s, 2H, NH_2_) and 6.40–8.89 (m, 7H, aromatic protons and pyridine-H). ^13^C-NMR (DMSO-d_6_): δ 22.97 (-CH_2_-CH_2_-), 29.23 [2(CH_2_)] attached to the aromatic ring of tetrahydronaphthalene), 107.17, 118.39, 124.78, 128.05, 128.42, 129.20, 129.56, 131.71, 137.98, 140.12, 140.68, 149.39, 153.11 (Ar-C), 163.88, 164.97 (2C=O). MS, *m/z* (%): (M^+^, 413, 14%).

*4-(2,6-Difluorophenyl)-6-(1,2,3,4-tetrahydronaphthalen-6-yl)-2-oxo-1,2-dihydropyridine-3-carboxamide* (**7b**). IR: (υ, cm^−1^) br. 3,380 (NH & NH_2_), 2,932 (CH_2_) and 1,666 (C=O). ^1^H-NMR (DMSO-d_6_): δ 1.69 (m, 4H, -CH_2_-CH_2_-), 2.71 (m, 4H, 2(CH_2_) attached to the aromatic ring of tetrahydronaphthalene), 3.34 (br. s, 2H, NH_2_), 6.55–8.72 (m, 7H, aromatic protons & pyridine-H) and 12.5 (NH). ^13^C-NMR (DMSO-d_6_): δ 22.98 (-CH_2_-CH_2_-), 29.18 (2(CH_2_) attached to the aromatic ring of tetrahydronaphthalene, 108.16, 111.59, 112.45, 118.37, 120.53, 123.07, 124.80, 128.41, 129.45, 130.15, 137.91, 140.51, 145.09, 148.75, 157.87, 159.81 (Ar-C), 163.20, 165.54 (2C=O). MS, *m/z* (%): (M^+^, 380, 23%).

#### 3.1.6. *Synthesis of 4-Aryl-N-(2',3',4',6'-tetra-O-acetyl-β-D-galactopyranosyl)-6-(1,2,3,4-tetrahydro-naphthalen-6-yl)-2-oxo-1,2-dihydro pyridine-3-carboxamides* **8a,b**

To a solution of **7a** and/or **7b** (0.001 mol) in acetone (10 mL), 2,3,4,6-tetra-*O*-acetyl-α-D-gluco- or galactopyranosyl bromide (4.10 g, 0.01 mol) was added. Few drops of acetic acid were added and the reaction mixture was heated with stirring on a water bath at 40–50 °C for 25 h. The reaction mixture was cooled and then poured onto ice water. The formed precipitate was washed for several times with water and recrystallized from ethanol (Table 1).

*4-(2,6-Dichlorophenyl-N-(2',3',4',6'-tetra-O-acetyl-β-D-galactopyranosyl)-6-(1,2,3,4-tetrahydronaphthalen-6-yl)-2-oxo)-1,2-dihydropyridine-3-carboxamide* (**8a**). IR: (υ, cm^−1^) ^1^H-NMR (DMSO-d_6_): δ 1.73 (m, 4H, -CH2-CH2-), 1.90–2.08 (4s, 12H, 4 × CH_3_CO), 2.75 [m, 4H, 2(CH_2_)] attached to the aromatic ring of tetrahydronaphthalene), 3.93 (m, 2H, 6'-H2) 4.08 (m, 1H, 5'-H), 4.20 (t, 1H, 4'-H) 5.15–5.17 (t, 2H, 3'-H & 2'-H), 5.35 (d, 1H, 1'-H), 6.42–8.99 (m, 7H, aromatic protons and pyridine-H) and 12.67 (NH). ^13^C-NMR (DMSO-d_6_): δ 19.85–20.04 (4 × -COCH3), 22.98 (-CH_2_-CH_2_-), 29.14 [2(CH_2_)] attached to the aromatic ring of tetrahydronaphthalene), 61.25 (CH_2_, C-6'), 65.88 (C-4'), 67.34 (C-2'), 72.1 (C-3'), 74.34 (C-5'), 80.23 (C-1'), 107.17–153.09 (Ar-C) and 163.87–190.99 (6 × CO). MS, *m/z* (%): (M^+^ +1, 744, 10.96).

*4-(2,6-Difluorophenyl-N-(2',3',4',6'-tetra-O-acetyl-β-D-galactopyranosyl)-6-(1,2,3,4-tetrahydronaphthalen-6-yl)-2-oxo)-1,2-dihydropyridine-3-carboxamide* (**8b**). IR: (υ, cm^−1^). ^1^H-NMR (DMSO-d_6_): δ 1.73 (m, 4H, -CH_2_-CH_2_-), 1.99–2.08 (4s, 12H, 4 × CH_3_CO), 2.74 (m, 4H, 2(CH_2_) attached to the aromatic ring of tetrahydronaphthalene), 3.92 (m, 2H, 6'-H2) 4.08 (m, 1H, 5'-H), 4.56 (t, 1H, 4'-H) 5.15–5.17 (t, 2H, 3'-H & 2'-H), 5.40 (d, 1H, 1'-H), 6.59–9.48 (m, 7H, aromatic protons and pyridine-H) and 12.66 (NH). ^13^C-NMR (DMSO-d_6_): δ 19.85–20.04 (4 × -COCH_3_), 22.98 (-CH_2_-CH_2_-), 29.14 (2(CH_2_) attached to the aromatic ring of tetrahydronaphthalene), 61.25 (CH_2_, C-6'), 65.88 (C-4'), 67.34 (C-2'), 72.1(C-3'), 74.34 (C-5'), 80.23 (C-1'), 108.17–159.79 (Ar-C) and 163.22–170.33 (6 × CO). MS, *m/z* (%): (M^+^, 710, 8.66).

### 3.2. Molecular Modelling

Autodock vina allows the flexible docking of ligands into their site of action. It has the ability to use all the rotable bonds of the ligands to give a number of conformations from which the best mode could be deduced. The crystal structure of tyrosine kinase was downloaded from protein data bank [28]. Pdb code = 1t46 was written in search area of the pdbsum by clicking on find tap the complexed inhibitor will be viewed showing all possible interactions with the binding site [29]. All compounds were built and saved as pdbqt format and docking with Autodock Vina was performed according to the specified condition in which the grid box was adjusted to have center x = 24.15, center_y = 22.8, and center_z = 12.9 by these centres the pocket with the main residues were involved inside the box.

### 3.3. *In Vitro* Cytotoxic Screening

#### 3.3.1. Tumor

Ehrlich Ascites Carcinoma (EAC) cells was kindly provided by the National Cancer Institute (Cairo University, Cairo, Egypt). The tumor line was maintained in female Swiss albino mice by weekly intra-peritoneal injection of 2.5 × 10^5^ cells/mouse according to the method recommended by the Egyptian National Cancer Institute, Cairo University. Such developed tumor is characterized by its moderate rapid growth which could not kill the animal due to the accumulation of ascites before about 14 days after transplantation. Cells were counted before injection using the bright line haemocytometer and dilutions made by physiological saline. The desired number of cells was injected in a volume of 0.5 mL/mouse. Solid Ehrlich carcinoma was induced by inoculation of 2.5 × 10^5^ cells in the back between the thighs of each animal [30].

#### 3.3.2. Animals

Adult female Swiss albino mice, obtained from the animal house of Cairo Cancer Institute (Cairo University, Egypt) weighing 18–22 g were used. The local committee approved the design of the experiments, and the protocol conforms to the guidelines of the National Institutes of Health (NIH). The animals were maintained on a standard pellet diet and tap water *ad libitum*.

#### 3.3.3. *In Vitro* Test for Cytotoxic Effect

A set of sterile test tubes were used, where 2.5 × 10^5^ tumor cells per ml were suspended in phosphate buffer saline, then 25, 50, 100 µg/mL of the test compound were added to the suspension, kept at 37 °C for 2 h. Trypan blue dye exclusion test was then carried out to calculate the percentage of non-viable cells [27].

## 4. Conclusions

Some substituted tetrahydronaphthalene (2,3,4,6-tetra-*O*-acetyl-β-D-gluco/-galactopyranosyl) derivatives were synthesized and tested for their anticancer activity. The preliminary *in vitro* cytotoxicity evaluation revealed that five compounds show promised activity. This encouraged us to do a molecular docking study to find out a suitable binding mode for these compounds. The docking was done against TK and the docking output was analyzed. The compounds have shown hydrogen bond formation with a reasonable distance ranging from 2.06 A° to 3.06 A° with Thr 670 and Cys 673 residues. No hydrogen bond was observed with neither Glu 640 nor Asp 810 residues as was expected from pdbsum. From that we can conclude that substituted tetrahydronaphthalene (2,3,4,6-tetra-*O*-acetyl-β-D-gluco/-galactopyranosyl) compounds may provide more activity by further study trying to modify their substitutions to give new derivatives with expected improved anticancer activity and that may be the subject of our future work.
